# Characteristics of a cohort of children with Juvenile Idiopathic Arthritis and JIA-associated Uveitis

**DOI:** 10.1186/s12969-015-0018-8

**Published:** 2015-06-02

**Authors:** Sheila T. Angeles-Han, Courtney McCracken, Steven Yeh, Kirsten Jenkins, Daneka Stryker, Kelly Rouster-Stevens, Larry B. Vogler, Scott R. Lambert, Carolyn Drews-Botsch, Sampath Prahalad

**Affiliations:** Department of Pediatrics, Emory University School of Medicine, 2015 Uppergate Drive, Atlanta, GA 30322 USA; Children’s Healthcare of Atlanta, 1405 Clifton Rd, Atlanta, GA 30329 USA; Department of Ophthalmology, Emory University School of Medicine, 1365-B Clifton Rd, Atlanta, GA 30322 USA; Rollins School of Public Health, Emory University, 1518 Clifton Rd, Atlanta, GA 30322 USA; Department of Genetics, Emory University School of Medicine, Atlanta, GA 30322 USA

**Keywords:** Juvenile idiopathic arthritis, Uveitis, Risk factors, Quality of life

## Abstract

**Background:**

Juvenile idiopathic arthritis-associated uveitis (JIA-U) can lead to poor visual outcomes and impact a child’s quality of life (QOL) and function. Our aim is to identify risk markers of JIA-U and examine differences in the QOL of children with JIA and JIA-U.

**Methods:**

Rheumatology and ophthalmology record reviews and questionnaires were completed every 4–6 months on 287 children with JIA. We collected arthritis, uveitis, and QOL data. We examined data through last study visit.

**Results:**

There were 52/287 (18 %) children with JIA-U who were younger at arthritis diagnosis, had oligoarticular persistent JIA, and ANA positive. Confirmed uveitis predictors were age at JIA diagnosis (OR = 0.86) and oligoarticular subtype (OR = 5.92). They had worse vision specific QOL and function, but similar general QOL. Blindness occurred in 17.5 % of children but was more common in African American children compared to non-Hispanic Caucasian children ((5/7 (71 %) vs. 2/29 (7 %), p <0.001) despite a similar uveitis prevalence (22 % vs. 16 %). Both races had similar complications, although band keratopathy was more frequent in African Americans (75 % vs. 15.6 %, p = 0.003).

**Conclusions:**

We confirm young age at JIA diagnosis and the oligoarticular JIA subtype as predictors of uveitis development. Although we were unable to identify predictors of ocular complications or blindness, AA children appeared to have a more severe disease course manifested by increased ocular complications, vision loss and blindness. Potential causes that warrant additional study include underlying disease severity, access to medical care and referral bias. Further investigation of the risk factors for vision-compromising uveitis and its’ long-term effects should be conducted in a large racially diverse population.

**Electronic supplementary material:**

The online version of this article (doi:10.1186/s12969-015-0018-8) contains supplementary material, which is available to authorized users.

## Background

Juvenile idiopathic arthritis-associated uveitis (JIA-U) can lead to ocular complications and permanent vision loss. Approximately 10-25 % of the 300,000 children in the United States with JIA develop uveitis within the first 4 years of their arthritis diagnosis [1–8]. JIA-U is an anterior, non-granulomatous chronic inflammatory ocular disease that is frequently asymptomatic at onset. Vision loss and ocular complications have been reported in 3-66 % of children [9, 6, 10–13, 3, 2]. These devastating ocular sequelae may be prevented by identifying children at greatest risk for severe disease.

The American Academy of Pediatrics guidelines recommend ophthalmology screening every 3–4 months in children with oligoarticular or polyarticular rheumatoid factor (RF) negative arthritis who are antinuclear antibody (ANA) positive, <7 years of age, and diagnosed with arthritis for ≤4 years since they are at high risk for developing uveitis [14]. These factors have been confirmed in several studies [14, 5, 15–17, 11, 9]. Risk factors for vision loss and/or ocular complications include male gender, short duration between arthritis and uveitis diagnoses, a uveitis diagnosis prior to arthritis diagnosis, young age at uveitis onset, an anterior chamber cell score ≥1+, initial visual acuity (VA) of 20/200 or worse, and presence of complications at first ophthalmology examination [18–21]. Hence, early detection of ocular disease and timely aggressive treatment in high risk patients can prevent poor visual outcomes.

Our primary objective is to identify risk markers for uveitis development and a severe ocular course in children with JIA by (1) comparing those with JIA alone and those with JIA-associated-uveitis (JIA-U), and (2) analyzing children with severe uveitis. We defined severe disease as a history of ocular complications, vision loss (VA 20/50 or worse) and legal blindness (VA 20/200 or worse).

## Methods

This is a retrospective analysis of prospectively collected data. It was approved by the Institutional Review Board of Emory University and conformed to the requirements of the US Health Insurance Portability and Privacy Act. Informed consent was obtained from the parent/legal guardian, and assent was obtained from the children if applicable. Research adhered to the tenets of the Declaration of Helsinki.

### Subjects

Eligible children presenting with a diagnosis of JIA or uveitis were enrolled from the pediatric rheumatology outpatient clinics from November 2011 to January 2015. They were followed prospectively from time of enrollment and returned for their usual follow up appointment every 3–6 months. Inclusion criteria included: 1) a diagnosis of JIA with or without uveitis and 2) English speaking. Exclusion criteria included: 1) a diagnosis of another chronic or autoimmune disease associated with uveitis; 2) major developmental disorders (i.e. cerebral palsy, autism); 3) other chronic disorders that can affect quality of life (QOL) and function; and 4) refusal to participate. Some patients were diagnosed with JIA or uveitis months or years prior to enrollment.

### Data collection

We reviewed rheumatology and ophthalmology medical records from time of presentation to every 3–6 months during the usual pediatric rheumatology follow up visit. Baseline data collected included date of birth, gender, parents self-described race and ethnicity, arthritis characteristics (onset, date of diagnosis, JIA subtype, joints with tenderness swelling and/or limitation, radiographs), uveitis characteristics (onset, date of diagnosis, laterality, location, ocular complications, surgeries), ocular exam (best corrected visual acuity (VA), intraocular pressure, anterior chamber cells and flare), labs (ANA, RF, erythrocyte sedimentation rate (ESR), HLA-B27, and anti-cyclic citrullinated peptide (anti-CCP)) and all medications used currently and in the past. Follow up data on arthritis, uveitis, labs and medications were collected at 3–6 month intervals (time of last study visit to current study visit) and during uveitis flares. Data from the ocular exam was recorded from every ophthalmology visit with varied intervals depending on presence of uveitis and disease activity.

### Measures of quality of life and function

We administered parent- and patient-based questionnaires at each study visit - the Childhood Health Assessment Questionnaire (CHAQ) to measure physical function, the Pediatric Quality of Life Inventory Version 4.0 (PedsQL) to measure overall QOL, and the Effects of Youngsters Eyesight on Quality of Life (EYE-Q) to measure vision related QOL and function [22–25]. Child-report forms were administered as appropriate per age based on the specific measure. Questionnaires were read by the study team to children who had significant visual impairment.

The CHAQ evaluates functional disability and has well-documented reliability and validity [22]. There are 20 questions encompassing 8 functional components: 1) dressing and grooming, 2) arising, 3) eating, 4) walking, 5) hygiene, 6) reach, 7) grip, and 8) activities. There are three parameters within each area: 1) difficulty in performing daily functions, 2) use of special aids or devices, and 3) activities that require assistance from another person. Scores range from 0 to 3 and lower scores indicate better physical function.

The PedsQL is a validated measure of health-related QOL in children and adolescents from 2 to 18 years of age [23]. Four core scales are measured on a 5-point-scale: 1) physical functioning (8 items), 2) emotional functioning (5), 3) social functioning (5), and 4) school functioning (5). Scores range from 0–100 with higher scores indicating better QOL. For this study, we included measures of overall QOL (PedsQL Total) and psychosocial related QOL (PedsQL Psychosocial).

The EYE-Q is a measure of vision related QOL and function in children [25, 24, 26]. It consists of both parent and patient self-reports for children 5–18 years old. There are 19 items that query on tasks that rely on distance, near, color, and night vision; functionality; and photosensitivity. QOL items inquire about feelings regarding medication use, missing school, and lab draws using a 5-point Likert scale response format. There is a question about ocular symptoms, the use of visual aids, and a subjective assessment of vision severity.

### Statistical analysis

Statistical analyses were conducted using SAS v9.3 for Windows (Cary, NC). Statistical significance was assessed at the 0.05 level unless otherwise noted. Data are summarized using means and standard deviations, medians and interquartile ranges (25^th^ – 75^th^ percentile) or counts and percentages, when appropriate. Duration of arthritis and/or uveitis were calculated as time between date of diagnosis to last study visit. Duration between uveitis and arthritis diagnosis was calculated by subtracting date of arthritis diagnosis from date of uveitis diagnosis. We compared children with JIA alone and JIA-associated uveitis using Chi-square tests and two sample t-tests. In instances of small expected cell counts (<5), exact Chi-square tests were used. When continuous data were skewed or non-normal, a nonparametric test (Mann Whitney-U or Kolmogorov-Smirnov test) was used in place of the two sample t-test.

We conducted further analysis in children with uveitis and compared those with and without ocular complications, those with a VA of 20/200 and worse, and African American (AA) and Caucasian (W) children with uveitis. Subgroups (i.e., complicated vs. uncomplicated, vision loss vs. no vision loss, AA vs. W) were compared using similar methodology described above. Multivariable logistic regression was used to simultaneously model multiple risk factors for development of uveitis. Similar models were constructed for the outcome of complicated disease. Candidate predictors identified in the univariate analyses were entered into the model. Stepwise backward elimination was used to obtain the final model. Non-significant variables were systematically removed until all variables in the model were significant at the 0.05 level or a significant reduction in model fit was observed.

## Results

### Demographics and clinical characteristics of cohort

There were 287 children with JIA of whom 52 (18 %) had uveitis at the last study visit (Table 1). Of the 52 children with uveitis, 5 with JIA alone developed uveitis after enrollment, and 1 child with uveitis alone developed JIA. Our cohort consisted of 71 % females, 77 % W, and 12 % AA children. The median (IQR) age of arthritis diagnosis was 6.4 years (2.9 – 11.7), and 41 % were of the oligoarticular persistent JIA subtype.

**Table 1:** Characteristics of Children with JIA and JIA-associated Uveitis

Characteristics	Group			
Median (25^th^ – 75^th^) unless otherwise specified	Overall (N = 287)	JIA (N = 235)	JIA-U (N = 52)	P-value
**Demographics**				
Gender, female, N (%)	205 (71.4 %)	165 (70.2 %)	40 (76.9 %)	0.332
Hispanic, N (%)	24 (8.4 %)	16 (6.8 %)	8 (15.4 %)	0.043*
Race, N (%)				
Caucasian	221 (77.0 %)	183 (77.9 %)	38 (73.1 %)	0.459
African American	36 (12.5 %)	28 (11.9 %)	8 (15.4 %)	0.500
Other	30 (10.5 %)	24 (10.2 %)	6 (11.5 %)	0.800
**Disease Characteristics**				
Age JIA diagnosis (yrs.)	6.4 (2.9 – 11.7)	7.7 (3.8 – 12.0)	2.8 (1.8 – 6.6)	<0.001*
Duration of JIA at last study visit (yrs.)	3.4 (1.8 – 5.9)	3.1 (1.6 – 5.3)	5.6 (2.9 – 8.9)	<0.001*
Total follow up time (months, mean ± SD)	15.1 ± 12	14.2 ± 11.7	18.8 ± 12.7	0.012*
JIA subtype, N (%)				
Oligo persistent	119 (41.1 %)	80 (34.0 %)	39 (75.0 %)	< 0.001*
Oligo extended	14 (4.9 %)	12 (5.1 %)	2 (3.9 %)	0.754
Polyarticular RF (−)	70 (24.4 %)	63 (26.8 %)	7 (13.5 %)	0.049*
Polyarticular RF (+)	13 (4.5 %)	13 (5.5 %)	0 (0.0 %)	0.135
Systemic	22 (7.7 %)	22 (9.4 %)	0 (0.0 %)	0.038*
Psoriatic	10 (3.5 %)	10 (4.3 %)	0 (0.0 %	0.217
Enthesitis related	37 (12.9 %)	33 (14.0 %)	4 (10.8 %)	0.260
Undifferentiated	2 (0.7 %)	2 (0.9 %)	0 (0.0 %)	1.00
**Labs, N (%)** ^**1**^				
ANA (+) (n = 286)	112 (39.3 %)	84 (35.9 %)	28 (54.9 %)	0.017*
RF (+) (n = 288)	26 (9.1 %)	26 (11.1 %)	0 (0.0 %)	0.013*
Anti-CCP (+) (n = 285)	24 (8.5 %)	24 (10.3 %)	0 (0.0 %)	0.020*
HLA-B27 (+) (n = 192)	32 (16.8 %)	26 (16.3 %)	6 (18.8 %)	0.740
Earliest ESR (n = 283)	16 (6 – 38)	14.5 (6 – 38)	22 (8 – 38)	0.327
**Medication Use, N (%) (n = 287)**				
Methotrexate oral	160 (55.9 %)	124 (52.8 %)	36 (70.6 %)	0.020*
Methotrexate injection	167 (58.4 %)	129 (54.9 %)	38 (74.5 %)	0.010*
Infliximab	38 (13.3 %)	21 (8.9 %)	17 (33.3 %)	< 0.001*
Etanercept	48 (17.0 %)	45 (19.4 %)	3 (5.9 %)	0.022*
Adalimumab	64 (22.4 %)	52 (22.1 %)	12 (23.5 %)	0.828
Abatacept	6 (2.1 %)	4 (1.7 %)	2 (3.9 %)	0.600
**Quality of Life Scores (child)** ^**a**^				
PedsQL^b^ Total	79.9 (66.5 – 90.2)	80.3 (66.5 – 90.2)	78.6 (66.2 – 90.7)	0.612
PedsQL^b^ Psychosocial	81.0 (67.2 – 90.3)	81.9 (69.4 – 90.5)	76.2 (65.0 – 89.7)	0.248
CHAQ^c^	0.25 (0.04 – 0.66)	0.25 (0.04 – 0.66)	0.25 (0.04 – 0.88)	0.690
EYE-Q^d^	3.62 (3.38 – 3.82)	3.68 (3.46 – 3.84)	3.35 (2.88 - 3.56)	< 0.001*
**Quality of Life Scores (parent)** ^**a**^				
PedsQL^b^ Total	81.7 (66.3 – 90.7)	81.8 (65.6 – 89.7)	81.3 (69.2 – 92.5)	0.679
PedsQL^b^ Psychosocial	82.5 (67.9 – 92.5)	81.2 (68.1 – 92.3)	82.4 (67.1 – 92.6)	0.933
CHAQ^c^	0.25 (0.04 – 0.63)	0.31 (0.06 – 0.67)	0.15 (0 – 0.49)	0.091
EYE-Q^d^	3.73 (3.48 – 3.86)	3.75 (3.55 – 3.88)	3.41 (3.20 – 3.64)	< 0.001*

^a^Indicates missing data; *p = <0.05; ^b^Pediatric Quality of Life Inventory; ^c^Childhood Health Assessment Questionnaire; ^d^Effects of Youngsters Eyesight on Quality of Life

Chi-square tests, two sample t-tests, non-parametric test (Mann Whitney-U or Kolmogorov-Smirnov test)

### Characteristics of children with uveitis

Children with uveitis were primarily female (77 %), and 73 % were W, 15 % were AA, and of these, 15 % were also Hispanic. Most had anterior uveitis (80 %), bilateral involvement (72 %) and several ocular complications (cataracts (31 %), synechiae (31 %), band keratopathy (25 %), glaucoma (17 %), and cystoid macular edema (15 %)). They were diagnosed with arthritis at a median of 2.8 years of age and uveitis at 4.8 years of age.

In comparing children with JIA alone vs those with JIA-U (Table 1), more children with JIA-U had oligoarticular persistent JIA (75 % vs. 34 %, p < 0.001), were significantly younger at arthritis diagnosis (2.8 vs. 7.7 years, p < 0.001), ANA positive (55 % vs. 36 %, p = 0.017), rheumatoid factor negative (0 % vs. 11 %, p = 0.013) and anti-CCP negative (0 % vs. 10 %, p = 0.020). They were less likely to be of the polyarticular RF negative (p = 0.049) and systemic JIA subtypes (p = 0.038).

On sub-analysis, we then considered 91 children who had JIA for ≥4 years since approximately 80 % of the cases of uveitis in children with JIA are diagnosed within 4 years of JIA diagnosis. Even after restricting the analyses to this subgroup, those with uveitis were still likely to be younger at arthritis diagnosis (p < 0.001), have oligoarticular persistent JIA (p <0.001), be RF negative (p = 0.026) and be anti-CCP negative (p = 0.026). The associations between ANA positivity, and the other JIA categories were no longer significant.

In comparing males and females with uveitis, females were younger at arthritis and uveitis diagnosis (2.3 years vs. 6.6 years, p = 0.002, and 4.5 years vs. 7 years, p = 0.017) and ANA positive (66.7 % vs. 16.7 %, p = 0.003). There were no differences in ocular complications or medication use.

Forty-five percent of children had vision loss, 50 % had at least one ocular complication, and 17 % (8/47) had a history of legal blindness. Of these 8 children, only one had a history of bilateral blindness. There were no significant differences in clinical characteristics based on the presence of mild vision loss (VA 20/50 or worse) (N = 26 vs. 20); however AA had more vision loss compared to W (6/7 (86 %) vs. 13/35 (37 %), p = 0.018) (data not shown).Vision loss was associated with higher rates of complications: cataract (p < 0.001), band keratopathy (p < 0.001), cystoid macular edema (p = 0.015) In addition, those with vision loss required the use of more anti-tumor necrosis factor agents (p = 0.042) . Comparing children with and without ocular complications (N = 26 vs. 26), those with complications had more blindness (0 % vs. 33.3 %, p = 0.002), and a history of steroid ocular injections (3.9 % vs. 36 %, p = 0.005). Children with a history of legal blindness (VA of 20/200 or worse) (N = 8) were primarily non-Hispanic (18 % vs. 0 %, p = 0.017), AA (62.5 % vs. 5.1 %, p < 0.001) and had significantly more ocular complications (i.e. cataracts (p < 0.001), glaucoma (p < 0.001), synechiae (p = 0.040), band keratopathy (p < 0.001), and cystoid macular edema (p < 0.001)), cataract extractions (p = 0.026), and intraocular steroid injections (p = 0.001).

### Predictors of uveitis development

On univariate analysis, oligoarticular persistent JIA subtype, ANA positivity, age at arthritis diagnosis, and anti-CCP negativity were potential predictors of uveitis development (Table 2). On multivariate analysis, age at arthritis diagnosis (OR = 0.91; 95 % CI [0.84 – 0.99] and oligoarticular JIA subtype remained significant (OR = 4.64; 95 % CI [2.21 – 9.75]) and were the best combination of predictors of uveitis among the models we considered (AUC = 0.77).

**Table 2:** Discriminatory Power of Single Risk Factors for JIA-U vs. Multiple Risk Factors

Model	Risk factor	Odds ratio	95 % CI	AUC
**Univariate Models**				
Oligoarticular Persistent Subtype	--	5.92	(2.99 – 11.74)	0.71
ANA Positivity	--	2.13	(1.15 – 3.92)	0.59
Age Arthritis Diagnosis (per 1 year increase)	--	0.86	(0.80 – 0.93)	0.71
AA Race vs. NHW	--	1.56	(0.65 – 3.74)	0.57
Anti-CCP negative	--	--	--	0.55
**Multivariable Models**	Risk Factor	Odds Ratio	95 % CI	AUC
Oligo. PE^a^ + Age Arthritis Diagnosis	Oligo PE^a^	4.64	(2.21 – 9.75)	0.77
	Age	0.91	(0.84 – 0.99)	
Oligo PE^a^ + ANA Positivity	Oligo PE^a^	5.30	(2.65 – 10.60)	0.73
	ANA +	1.65	(0.86 – 3.15)	
Oligo PE^a^ + Large Joints	Oligo PE^a^	5.39	(2.64 – 11.01)	0.72
	Large Joints	1.53	(0.62 – 3.79)	
Age Arthritis Diagnosis + ANA Positivity	Age	0.87	(0.81 – 0.94)	0.71
	ANA +	2.05	(1.08 – 3.90)	
Age Arthritis Diagnosis + ANA Positivity + Oligo PE^a^	Age	0.92	(0.85 – 1.00)	0.76
	ANA +	1.82	(0.94 – 3.54)	
	Oligo PE^a^	4.17	(1.97 – 8.83)	

^a^Oligoarticular Persistent JIA subtype; Multivariable logistic regression

### Quality of life and function

Children with uveitis had worse vision related QOL and function compared to children with JIA alone as measured by the EYE-Q child and parent reports (p < 0.001) (Table 1). However, all children had similar overall QOL, psychosocial QOL and physical function as measured by the PedsQL and CHAQ.

### Medication use

Most children with uveitis were treated with subcutaneous and/or oral methotrexate (85 %). Of the 8 patients who had never been on methotrexate (15 %): 1 has only used NSAIDS; 6 have taken NSAIDs and steroid eye drops; and 1 has only taken steroid eye drops. For those who were refractory to methotrexate, 33 % of children with uveitis were treated with infliximab and 24 % were treated with adalimumab. Children with vision loss and blindness were treated mainly with infliximab (p = 0.014, p = 0.022, respectively). There was no significant difference in the use of methotrexate, infliximab, etanercept, adalimumab or abatacept in children with and without ocular complications, or in AA and W children with uveitis. All children may have been on ≥1 medication.

Of the 44 children treated with methotrexate and/or a biologic, 12 (27 %) developed uveitis during treatment. Of the 12, 10 developed uveitis while on methotrexate, 1 developed uveitis after discontinuing methotrexate (within 6 months) but on abatacept, and 1 while on both methotrexate and adalimumab. The majority (70 %) started methotrexate after their uveitis diagnosis.

### Epidemiology of uveitis

In our 52 children with uveitis, we had data on time to uveitis diagnosis in 50 patients in relation to arthritis diagnosis (Table 3). Approximately 24 % of children were diagnosed with uveitis prior to JIA, and 22 % within the 1^st^ year of their JIA diagnosis. Cumulatively, 86 % of children were diagnosed with uveitis within 4 years of their JIA diagnosis, and 4 % by 5 years.

**Table 3:** Uveitis diagnosis in relation to Arthritis JIA diagnosis

Diagnosis time	N^a^ = 50	Cumulative %
Prior to JIA Diagnosis	12 (24.0 %)	12 (24.0 %)
Within 1 year of JIA Diagnosis	11 (22.0 %)	23 (46.0 %)
Within 2 years of JIA Diagnosis	10 (20.0 %)	33 (66.0 %)
Within 3 years of JIA Diagnosis	5 (10.0 %)	38 (76.0 %)
Within 4 years of JIA Diagnosis	5 (10.0 %)	43 (86.0 %)
Within 5 years of JIA Diagnosis	4 (8.0 %)	47 (94.0 %)
More than 5 years	3 (6.0 %)	50 (100 %)

^a^N = 2 Missing

### Children with uveitis compared by race

In examining our data on the 8 children with legal blindness (VA of 20/200 or worse), we noted an increased frequency of AA children (5/7 (71 %) with a blind outcome compared to W children (2/29 (7 %)) despite a similar uveitis prevalence (22 % vs. 16 %) when restricting to non-Hispanic Caucasians and excluding children with missing data (Table 4). Although our results on race are underpowered due to a small number of AA children with uveitis, we conducted sub-analysis to compare AA and W race since the course of AA children with uveitis has rarely been explored. AA children were significantly older at uveitis diagnosis (11.3 years vs. 4.6 years, p = 0.033), and had an increased history of a VA of 20/200 or worse (71.4 % vs. 6.9 %, p < 0.001) and band keratopathy (75 % vs. 15.6 %, p = 0.003). They appeared to have more ocular complications, and upon further examination, AA had on average 3 times as many complications as NHW (p < 0.001). There were no differences in insurance status, age at arthritis diagnosis, JIA subtypes, duration between uveitis and arthritis diagnoses, treatment, or QOL.

**Table 4:** Comparison of African American and Caucasian Children with JIA-associated Uveitis

Characteristics	Race		P-value
Median (25^th^ – 75^th^) unless otherwise specified	AA (N = 8)	NHW (N = 32)	
**Demographics**			
Age at last study visit	16.3 (10.0 – 17.5)	8.5 (6.7 – 12.4)	0.024
Gender, female, N (%)	5 (62.5 %)	24 (75.0 %)	0.660
Insurance- Private, N (%)	3 (37.5 %)	21 (66.6 %)	0.147
**JIA Disease Characteristics**			
Age at arthritis diagnosis (yrs.)	3.4 (2.5 – 16.5)	2.8 (1.7 – 5.0)	0.172
Duration of JIA at last study visit (yrs.)	5.7 (2.0 – 14.0)	5.6 (3.0 – 8.9)	0.881
JIA Subtype, N (%)			
Oligoarticular persistent	4 (50.0 %)	26 (81.3 %)	0.089
Polyarticular rheumatoid factor (−)	3 (37.5 %)	4 (12.5 %)	0.128
Enthesitis related arthritis	1 (12.5 %)	2 (6.3 %)	0.498
**Uveitis Disease Characteristics**			
Age at uveitis diagnosis (yrs.)	11.3 (4.6 – 14.5)	4.6 (3.4 – 6.8)	0.033*
Duration of uveitis at last study visit (yrs.)	4.4 (3.2 – 7.2)	3.4 (1.3 – 7.1)	0.395
Duration between uveitis and arthritis diagnosis (yrs.)	1.3 (−1.2 – 4.0)	1.3 (0 – 2.4)	0.970
Location of disease, N (%)^a^			1.00
Anterior	5 (83.3 %)	23 (79.3 %)	
Unknown	1 (16.7 %)	6 (20.7 %)	
Bilateral disease, N (%)^a^	6 (75.0 %)	19 (70.4 %)	1.00
Visual acuity worse than 20/200	5 (71.4 %)	2 (6.9 %)	0.001
**Type of Complications, N (%)**			
Cataracts	5 (62.5 %)	9 (28.1 %)	0.102
Glaucoma	3 (37.5 %)	4 (12.5 %)	0.128
Synechiae	4 (50.0 %)	8 (25.0 %)	0.211
Band keratopathy	6 (75.0 %)	5 (15.6 %)	0.003*
Cystoid macular edema	3 (37.5 %)	4 (12.5 %)	0.128
Other complications	3 (37.5 %)	3 (9.4 %)	0.082
**Ocular Surgeries, N (%)**			
Cataract extraction	1 (12.5 %)	2 (6.3 %)	0.498
Steroid ocular injection	2 (25.0 %)	7 (21.9 %)	1.00
**Labs, N (%)** ^**a**^			
ANA	4 (50.0 %)	18 (56.3 %)	1.00
HLA-B27	0 (0 %)	5 (25.0 %)	0.298
**Medication Use, N (%)** ^**a**^			
Methotrexate oral	4 (50.0 %)	22 (71.0 %)	0.402
Methotrexate subcutaneous injection	6 (75.0 %)	22 (71.0 %)	1.00
Infliximab	4 (50.0 %)	9 (29.0 %)	0.402
Etanercept	0 (0 %)	3 (9.7 %)	1.00
Adalimumab	1 (12.5 %)	8 (25.8 %)	0.653
Abatacept	0 (0 %)	1 (3.2 %)	1.00
**Quality of Life Scores (child)** ^**a**^			
PedsQL^b^ Total	73.1 (54.1 – 82.8)	77.5 (67.4 – 90.8)	0.416
PedsQL^b^ Psychosocial	69.6 (56.0 – 83.1)	76.2 (65.0 – 90.0)	0.396
CHAQ^c^	0.27 (0.15 – 0.84)	0.38 (0.04 – 0.88)	0.875
EYE-Q^d^	2.9 (2.3 – 3.6)	3.3 (3.0 – 3.5)	0.480
**Quality of Life Scores (parent)** ^**a**^			
PedsQL^b^ Total	69.1 (46.9 – 83.9)	80.3 (71.8 – 92.6)	0.145
PedsQL^b^ Psychosocial	64.3 (51.9 – 80.8)	84.2 (68.2 – 92.6)	0.061
CHAQ^c^	0.51 (0.0 – 1.28)	0.18 (0.0 – 0.45)	0.367
EYE-Q^d^	2.9 (2.0 – 3.6)	3.4 (3.2 – 3.6)	0.189

^a^Indicates missing data; *p = <0.05; ^b^Pediatric Quality of Life Inventory; ^c^Childhood Health Assessment Questionnaire; ^d^Effects of Youngsters Eyesight on Quality of Life

Chi-square tests, two sample t-tests, non-parametric test (Mann Whitney-U or Kolmogorov-Smirnov test)

Table 5 examines the characteristics of the 8 AA children with uveitis based on age of JIA diagnosis. Four children were diagnosed with JIA at <7 years of age (AAP cut off age for frequent ocular examinations) of whom three were diagnosed with uveitis within 4 years of arthritis. They had a history of a VA of worse than 20/200 and ocular complications. The four that were diagnosed with JIA ≥7 years of age had uveitis either at presentation or prior to a JIA diagnosis and three had better VA and less complications. The one child diagnosed at 7 years of age had disease that was similar to the younger group, and it is possible she had an earlier onset that was unrecognized.

**Table 5:** Characteristics of Children of African Descent with JIA-associated Uveitis Based on Age at JIA diagnosis

Patient	Sex	Age at JIA Diagnosis (years)	Age at Uveitis Diagnosis (years)	Time Between JIA and Uveitis Diagnosis (years)^a^	JIA Subtype	ANA	Uveitis Laterality-Location	Worst Visual Acuity	Complications
1	F	2.0	4.6	2.6	Oligo^b^ Persistent	+	Bilateral- Anterior	20/400	Synechiae, band keratopathy, macular edema, cataracts
2	F	2.5	13.3	10.8	Oligo^b^ Persistent	+	Bilateral- Anterior	20/2000 Counting Fingers	Glaucoma, band keratopathy, macular edema, aphakia
3	F	2.8	4.0	1.2	Oligo^b^ Persistent	+	Bilateral-Anterior	20/300	Band keratopathy, ocular hypertension, cataracts, synechiae, macular edema
4	M	3.4	7.4	4.0	Poly^c^ RF (−)	-	Bilateral-Anterior- Intermediate	20/800	Cataracts, synechiae, band keratopathy
5	F	7.0	7.0	0	Poly^c^ RF (−)	-	Left-Anterior	20/4000 Hand motion	Cataracts, glaucoma, band keratopathy
6	M	14.5	14.5	0	ERA^d^	-	Bilateral – Anterior	20/50	None
7	F	16.5	11.3	−5.2	Oligo^b^ Persistent	+	Right- Anterior	20/40	Cataracts, synechiae, band keratopathy
8	M	18.4	17.2	−1.2	Poly^c^ RF (−)	-	Bilateral – Unknown	Unknown	None

^a^Negative numbers indicate diagnosis of uveitis prior to JIA; ^b^oligoarticular; ^c^polyarticular; ^d^enthesitis related arthritis

## Discussion

Our study confirms known risk factors associated with uveitis development in children with JIA– young age at JIA diagnosis, oligoarticular JIA subtype, ANA positivity, RF negativity and anti-CCP negativity; only the oligoarticular JIA subtype and young age at arthritis diagnosis remained significant predictors in our model [6, 27, 12]. When analysis was restricted to children at lower risk for uveitis (a diagnosis of JIA >4 years), these two predictors remained significant. We also confirm reports that most children are diagnosed within the first 4 years of arthritis (86 %), hence regular screening within the first 4 years of a JIA diagnosis is crucial. We were unable to identify predictors of severe uveitis (increased ocular complications, blindness) and found no significant differences in gender, duration between arthritis and uveitis diagnoses, age at uveitis onset, and a uveitis diagnosis prior to arthritis diagnosis in children with severe disease.

Uveitis can have a negative impact on a child’s daily function and ability to perform visual tasks in the home and school [28]. Additionally, children need regular ophthalmology and rheumatology physician visits, phlebotomy, frequent ophthalmic drops, and systemic immunosuppressive therapy consisting of oral medications, injections and infusions. Investigation into the effects of uveitis on a child’s QOL and function is important. Most studies use general QOL measures, arthritis specific measures, and the ocular exam as a measure of outcome. The EYE-Q is the only patient reported outcome measure specific to uveitis which we are currently validating in a pediatric uveitis population [24, 25]. In our cohort, children with uveitis appeared to suffer from worse vision specific function and QOL but no differences were apparent in general QOL or physical function. We expected those with both JIA and uveitis to experience worse general QOL due to involvement of two systems. It is possible that any diagnosis of chronic autoimmune disease affects QOL equally since care and treatment are similar, irrespective of number of diagnoses and disease activity. This emphasizes the importance of a global and comprehensive assessment of outcomes that takes into consideration vision specific measures, and not limited to general measures.

Race can be associated with various autoimmune conditions, including JIA and systemic lupus erythematosus. In JIA, more children of European descent develop oligoarticular disease, whereas non-European children develop polyarticular rheumatoid factor (RF) positive JIA with worse arthritis outcomes [29–31]. Since JIA subtypes differ in their association with uveitis, and race predisposes to different JIA categories, race may influence uveitis risk.

Few studies describe the association of race with pediatric uveitis. In adults, the Pacific Ocular Inflammation Study noted that of 224 uveitis cases, 38 % were Asian, 37 % were white, and only 2 % were Black [32]. However, Blacks had the highest incidence of uveitis at 39.6 cases per 100,000 patient years. In a JIA cohort in Toronto, the incidence of uveitis was similar in children of European and non-European ancestry (14.4 % vs. 12.8 %) although when compared to the general Toronto population, risk was increased in European children and decreased in non-European ancestries [29].

Uveitis has been reported in approximately 6-8 % of AA children with JIA and ANA positivity varies although other reports no associations with race (Table 6) [33, 34, 29, 16, 35]. In our cohort, uveitis frequency was similar in AA and W children (22 % vs. 17 %), with only 4 AA children who were ANA positive. This contrasts with our findings in the large Childhood Arthritis and Rheumatology Research Alliance (CARRA) registry (6 % vs. 12 %) which consisted of almost 4000 children with JIA [16]. However, our population may differ due to our location in the Southeast leading to ascertainment bias.

**Table 6:** Comparison of Uveitis Frequency in Various African American (AA) Juvenile Arthritis Population Studies

Study	Total children with juvenile arthritis	% (N) of AA with juvenile arthritis	% (N) of AA with uveitis	% (N) of aa with uveitis who are ana (+)
Haffejee, 1984 [36]	60 (systemic, oligo^a^ and poly^b^)	100 % (60)	8.3 % (5)	0 %
Schwartz, 1997 [34]	172 (oligo^a^ and poly^b^)	20 % (35)	8.7 % (3)	0 %
Adelowo, 2010 [37]	23 (systemic, oligo^a^ and poly^b^)	100 % (23)	8.6 % (2)	12.4 % (1/8, unknown if uveitis)
Angeles-Han, 2013 [16]	3967 (all JIA)	5.6 % (220)	6 % (13)	44 % (4)
Current cohort	287 (all JIA)	13 % (38)	21 % (8)	50 % (4)

^a^oligoarticular; ^b^polyarticular

A 1984 study demonstrated that 8.3 % of 42 South African children with JIA developed uveitis, but none were ANA positive [36]. In 1997, of 172 children with juvenile rheumatoid arthritis (JRA), 20 % were of African descent, 8.7 % of these had uveitis, and none were ANA positive [34]. In 2007, 4 % of 758 children with JIA were of African descent and had a lower risk of developing JIA-U [29]. In 2010, Adelowo examined 23 children with JIA and noted that only two (8.6 %) were diagnosed with uveitis [37]. In 2013, we reported that only 13 of the 220 (5.6 %) AA children in the Childhood Arthritis and Rheumatology Research Alliance (CARRA) registry developed uveitis and 4 of them were ANA positive [16]. Hence, only small cohorts of AA children with JIA and uveitis have been examined.

There is less information on the uveitis course of AA children with JIA, and one study describes severe outcomes in African children with uveitis secondary to various etiologies (idiopathic, Still’s disease, and sympathetic ophthalmia) [38]. Woreta et. al. report that in their JIA-U cohort, there were no associations between race and ocular complications, a VA of 20/50 or worse, or a VA of 20/200 or worse, although they only had 5 blacks and 64 white patients [39]. In our cohort, AA and W children had similar arthritis characteristics since all AA children were primarily of the oligoarticular or polyarticular RF negative JIA subtype, and uveitis was diagnosed within the first 4 years of JIA. However, AA children were diagnosed at an older age and suffer from more ocular complications, vision loss, and blindness. Examining the characteristics of each AA child with JIA-U, we note that those diagnosed with JIA at 7 years and younger had a more severe course, hence, the AAP recommended screening guidelines are crucial and more frequent monitoring may be important (Table 5). We are unable to ascertain whether this is secondary to biologic differences or variances in access to health care. This needs further study in a large racially diverse multi-center population. There are otherwise no other reports on the course of disease in children of African descent.

### Strengths and limitations

Our current cohort may not accurately reflect uveitis prevalence since not all children have had JIA for 4 years and may still develop uveitis. Thus, longer follow up is needed, especially in young children with early disease since they are at highest risk for uveitis. We continue to follow our cohort prospectively and recruit patients with newly diagnosed JIA.

Exploration of the association of race with uveitis should be performed in a larger and more diverse cohort, likely in a multi-center study. There appeared to be differences in uveitis course based on race, but our analysis was underpowered. However, although our cohort of AA children is small, the number of those with JIA-U is similar to previously published reports, including our analysis of the large CARRA registry which had 13 (6 %) AA children with uveitis (Table 5). We had an increased frequency of uveitis compared to other studies, and this may be due to our institution being a tertiary center in the Southeast where there is a larger AA population. A report of uveitis prevalence in adults in the Southeastern US in 1997 demonstrated that of 385 patients, 31 % were African American which is also of increased frequency [40]. Focusing on regions with diverse populations could help us better appreciate the differences in outcomes associated with race, and our results suggests the need for further evaluation.

We were unable to make conclusions on the role of Hispanic ethnicity since non-English speaking patients were excluded. However, we have translated all our instruments and are actively recruiting children from this population.

## Conclusions

In conclusion, our results confirm age at JIA diagnosis and the oligoarticular JIA subtype as predictors of uveitis development, but we were unable to confirm predictors of severe disease. We demonstrate that visual disability may be underestimated in JIA outcome studies since few studies look at vision specific QOL and function in children with uveitis. Vision specific measures may reveal areas of vision that are not quantified by general measures or the ocular exam. A comprehensive assessment of disability that incorporates uveitis specific measures may be a more clinically sensitive assessment of visual outcome.

Our study also highlights the potential role of race in uveitis and the need for further exploration in a large racially diverse cohort, likely in a multi-center study. Identifying high risk children will enable early, aggressive treatment and potentially prevent visual complications associated with severe disease.
